# Quantum-Gravity Stochastic Effects on the de Sitter Event Horizon

**DOI:** 10.3390/e22060696

**Published:** 2020-06-22

**Authors:** Claudio Cremaschini, Massimo Tessarotto

**Affiliations:** 1 Research Center for Theoretical Physics and Astrophysics, Institute of Physics, Silesian University in Opava, Bezručovo nám.13, CZ-74601 Opava, Czech Republic; maxtextss@gmail.com; 2 Department of Mathematics and Geosciences, University of Trieste, Via Valerio 12, 34127 Trieste, Italy

**Keywords:** covariant quantum gravity, cosmological constant, de Sitter space-time, event horizon, stochastic effects, Hawking temperature

## Abstract

The stochastic character of the cosmological constant arising from the non-linear quantum-vacuum Bohm interaction in the framework of the manifestly-covariant theory of quantum gravity (CQG theory) is pointed out. This feature is shown to be consistent with the axiomatic formulation of quantum gravity based on the hydrodynamic representation of the same CQG theory developed recently. The conclusion follows by investigating the indeterminacy properties of the probability density function and its representation associated with the quantum gravity state, which corresponds to a hydrodynamic continuity equation that satisfies the unitarity principle. As a result, the corresponding form of stochastic quantum-modified Einstein field equations is obtained and shown to admit a stochastic cosmological de Sitter solution for the space-time metric tensor. The analytical calculation of the stochastic averages of relevant physical observables is obtained. These include in particular the radius of the de Sitter sphere fixing the location of the event horizon and the expression of the Hawking temperature associated with the related particle tunneling effect. Theoretical implications for cosmology and field theories are pointed out.

## 1. Introduction

Cosmological solutions for the space-time metric tensor predicted by the Einstein field equations (EFE) of classical general relativity (GR, [1,2]) provide mathematical solutions to a variety of astrophysical and physical scenarios, which include for example cosmological theories of galaxy evolution [3], compact object formation of primordial origin [4,5,6,7], particle physics phenomenology in connection with inflationary models [8,9], the nature of dark matter and dark energy [10,11,12,13], and the geometric structure of the universe and its dynamical history [14,15,16,17].

However, a distinctive property of classical and even quantum gravity solutions is related to coordinate singularities in the line-element representation of the metric tensor. In particular, this implies the occurrence of event horizons that exhibit typically a deterministic character and are effectively considered as space-time frontiers, i.e., which separate “incommunicable” regions of space-time having different metric signatures. The same feature, for example, is characteristic of cosmological solutions, such as the Big Bang singularity in the case of the Friedman–Robertson–Walker field tensor, and of event horizon surfaces separating domains of space-time, like the de Sitter vacuum solution. This means that the radii of the corresponding event horizons are usually considered to be prescribed as deterministic quantities. The basic consequence is that regions with different metric signatures are regarded as impenetrable not only for classical matter and radiation, but also for quantum particles and fields. In this regard, the so-called black hole no-hair theorems have an emblematic character [18]. Even if such theorems may be a very good approximation as far as the macroscopic behavior of a black hole is concerned, at the quantum level, it may be a whole different story. In fact, in quantum gravity, the very idea of a deterministic event horizon appears not only counter-intuitive, but even absurd. Notice that here, we are not specifically referring to Planck-length, minimal-length theories or discrete theories like loop quantum gravity, because the same issue may be naturally set also for continuum and manifestly-covariant theories of quantum gravity.

In this reference, additional challenging questions include the role that event horizons undertake in classical and quantum descriptions of general relativity, the nature of their existence as space-time frontiers and their mathematical description, and the inspection of quantum physical and thermodynamics phenomena that can occur in their surroundings as a consequence of interaction with particles and fields, including the gravitational field itself [19,20,21,22,23]. In fact, event horizons may be characterized by the occurrence of energetic particle phenomena and collective interactions, and for this reason, they are also expected to provide the background for quantum field phenomena, which may ultimately involve also the dynamics of the same gravitational field [24,25,26], the physics of event horizons [27], as exemplified by particle emission and acceleration mechanisms [28], and phenomena related to entropy creation/conservation [29,30,31,32].

In such a context, therefore, the conjecture arises whether stochastic, i.e., quantum effects, in the prescription of an event-horizon surface belonging to the four-dimensional space-time, might actually locally arise. In particular, if confirmed, this means that a new kind of quantum (or even in some sense “classical”, i.e., occurring on finite space-time scale lengths) tunneling effect might occur, since in such a case, a particle might have a finite probability density of being at the same time either “in” or “out” with respect to a stochastic surface, which is no-longer prescribed as a deterministic barrier. The basic issue is therefore whether, and eventually how, such a type of stochastic effect might be included in a continuum theory of quantum gravity based on a manifestly-covariant theory and how they might affect, in turn, cosmological solutions of the space-time metric tensor predicted by the Einstein field equations, with particular reference to cosmological solutions, which are typically characterized by a non-vanishing cosmological constant [33,34,35].

In this paper, the issues of the possible stochastic character of the cosmological constant and, consequently, of the analogous stochastic property of the de Sitter event horizon are analyzed. There are several different qualitative physical motivations behind such a conjecture:

- The first one is related to the physical origin of the cosmological constant and whether an intrinsic stochastic, i.e., quantum, effect might also affect its nature.
- The second reason is that, as stated above, from the point of view of quantum gravity, the absolute separation surfaces of space-time, such as the de Sitter event horizon, are meaningless or expected to be affected by quantum effects of a stochastic nature.
- The third physical motivation is that in quantum gravity, “interior” and “exterior” parts of event horizons, as in the case of the de Sitter space-time solution considered here, should not be treated as absolutely incommunicable. In other words, suitable quantum tunneling effects might arise for massive particles (such as gravitons), possibly permitting quantum transitions between these domains.
- The fourth reason is that the realization of event horizons as physical phenomena cannot be absolutely stable (this is unreasonable due to the obvious fact that any state of matter and consequently of space-time is not eternal) and can exhibit a stochastic behavior.

The conclusion follows by investigating the indeterminacy properties of the probability density function associated with the quantum gravity state of massive gravitons in the context of the axiomatic formulation of quantum gravity based on the theory of manifestly-covariant quantum gravity (CQG theory) developed recently [36,37,38]. This provides a novel route to the establishment of a self-consistent theory of quantum gravity underlying the standard formulation of general relativity. The new formalism satisfies simultaneously the fundamental principles of quantum mechanics and quantum field theory, like the probabilistic interpretation built into their conceptual structure [39], as well as the principles of covariance and manifest covariance at the basis of the classical Einstein theory of GR [40]. In GQC theory, this feature is realized by adopting a space-time representation in terms of superabundant variables, whereby manifest covariance is defined with respect to a continuum background metric tensor $\hat{g}\equiv \left\{{\hat{g}}_{\mu \nu }\right\}$, which raises/lowers tensor indices and defines the geometric properties of the space-time, while the quantum gravitational field $g\equiv \left\{g_{\mu \nu }\right\}$ encoding its quantum properties evolves dynamically over $\hat{g}$ according to a defined quantum wave equation (CQG-wave equation) [37,38]. The latter follows from the identification of the Hamiltonian structure associated with the classical space-time, with the prescription of the corresponding manifestly covariant Hamilton equations [36] and the related Hamilton–Jacobi theory [41] being obtained in the framework of a synchronous variational principle [42]. The implementation of standard quantization methods to the Hamiltonian structure of GR then leads to the realization in four-scalar form of the CQG-wave equation for the corresponding CQG-state and wave function ψ. Remarkably, the CQG-wave equation realizes a Schroedinger-like hyperbolic first-order partial differential equation with respect to an invariant proper-time parameter *s*, which parametrizes the temporal evolution of the gravitational quantum state. In agreement with the covariance principle, such a parameter is defined with respect to the background metric tensor $\hat{g}$ as the proper-time associated with suitable classical sub-luminal geodesic trajectories, namely through the differential identity $ds^{2}={\hat{g}}_{\mu \nu }dr^{\mu }dr^{\nu }$ (see the discussion in [37]). It must be stressed that, as a consequence of manifest covariance, both classical and quantum Hamiltonian representations are cast in four-tensor frame-independent forms while the quantum probability density ${\left|\psi \right|}^{2}$ acquires the character of a physical observable and therefore necessarily coincides with a four-scalar. The CQG theory implies, in particular, the validity of four-tensor quantum Hamilton equations, to be intended as quantum hydrodynamic equations associated with the quantum wave function ψ and prescribed in terms of a Hamiltonian hydrodynamic state $x=\left(g^{\mu \nu },\Pi_{\mu \nu }\right)$, where $g^{\mu \nu }$ and $\Pi_{\mu \nu }$ denote independent four-tensor canonical variables identified respectively with the generalized Lagrangian coordinates and conjugate canonical momenta.

The characteristic formalism of the manifestly-covariant representation of CQG theory permits the development of a hydrodynamic interpretation of quantum gravity, in analogy with the hydrodynamic representation of quantum mechanics [43]. The starting point in this direction is the adoption of the Madelung representation for the quantum wave function and the consequent realization of the CQG-wave equation in terms of corresponding quantum hydrodynamic equations, which, as such, are natively expressed in Eulerian form [44,45,46]. The notable feature is that a corresponding Lagrangian representation can equivalently be reached for the same hydrodynamic equations based on a stochastic trajectory-based representation of CQG theory. As shown in [47], this representation is based on a covariant generalized Lagrangian path (GLP) approach relying on a suitable statistical representation of Bohmian Lagrangian trajectories. The formalism is analogous to the one developed earlier for non-relativistic quantum mechanics [48], whereby each Bohmian trajectory is replaced by a family of stochastic trajectories carrying a given probability density function. An important advantage of the GLP formalism in the present context is that it affords the analytical construction of generally non-stationary solutions for the CQG-wave equation with a non-vanishing cosmological constant that are non-dispersive in proper-time and exhibit Gaussian-like probability densities [47,49].

Based on these premises, in the present research, we intend to address the theoretical problem of investigating stochastic phenomena induced by quantum gravity effects in the framework of CQG theory, which can characterize, in particular, physical observables of the cosmological de Sitter solution. Starting from the treatment initiated in [47,49,50,51], the issue pertains first to the determination of the stochastic character of the cosmological constant arising from the non-linear quantum-vacuum Bohm interaction as predicted by CQG theory. This feature follows by investigating the quantum hydrodynamic equations characteristic of CQG theory. In fact, when cast in terms of the stochastic trajectory-based representation referred to as the generalized Lagrangian path (GLP) [47], an explicit stochastic realization of the quantum probability density function (PDF), in terms of an appropriate four-scalar Gaussian-like function, is made possible. In such a representation, the PDF depends explicitly on a stochastic tensor $\Delta g_{\mu \nu }$ representing the fluctuations of the quantum field tensor $g_{\mu \nu }$. However, as we intend to show here, the same analytical representation is actually non-unique. Thus, generalizing the functional dependence considered in [47], besides the stochastic tensor $\Delta g_{\mu \nu }$, the same PDF may depend also on an additional stochastic parameter α. In fact, in the context of CQG theory, there is a precise technical reason for the occurrence of the new stochastic effect. This is due to the arbitrariness in the prescription of the tensor displacement field, which enters in the theory ($\Delta g_{\mu \nu }$), which can be now replaced with the stochastic displacement field:

(1) $$\Delta g_{\mu \nu }\left(\alpha \right)=\Delta g_{\mu \nu }+\alpha {\hat{g}}_{\mu \nu },$$

with $\alpha \in \left[\alpha_{0},\alpha_{1}\right]\subseteq R$ being a real stochastic parameter and ${\hat{g}}_{\mu \nu }$ a suitable background space-time field tensor. Notice that here, the support of the stochastic parameter α should be ordinarily considered bounded to warrant the strict positivity of the stochastically-modified cosmological constant to be determined later. The result is based on the construction of the corresponding modified form of the stochastic quantum-modified Einstein field equations, in which the new quantum stochastic term can be shown to contribute to the same equations by means of a symmetric second-order tensor. A realization of such a tensor based on a comparison with the stress-energy tensor of a perfect fluid allows for the physical interpretation of the quantum corrections to EFE and, at the same time, permits proving the existence of a modified cosmological de Sitter solution for the same metric and curvature tensors, characterized by a stochastically-modified cosmological constant. The second task deals, in fact, with investigating precisely how the new stochastic quantum PDF affects and modifies the cosmological constant and thus possibly determines also new physical properties of the de Sitter event horizon itself. The treatment of these new stochastic quantum gravity phenomena is investigated by the calculation of stochastic averages of physical observables. These include in particular the stochastically-averaged radius of the de Sitter sphere, which fixes the location of the de Sitter event horizon, and the expression of the Hawking temperature associated with it. A main achievement of the study is the proof that the stochastic behavior of quantum gravity can affect in a non-trivial way the thermodynamic description of the continuum gravitational field and of the related particle tunneling effect that can arise across the horizon boundary, with relevant implications both for cosmology and field theories.

## 2. Theory of Manifestly-Covariant Quantum Gravity

In this section, we recall the theoretical framework of the theory of manifestly-covariant quantum gravity (CQG theory) in its trajectory-based representation, in turn based on the generalized Lagrangian path (GLP) formalism. More precisely, the emphasis here is its connection with the so-called quantum-modified Einstein field equations (EFE), which determine the continuum background metric tensor $\hat{g}\equiv \left\{{\hat{g}}_{\mu \nu }\right\}$. As pointed out earlier in [49,50] in the framework of CQG theory, its construction is based on suitable rigorous, but also physically-intuitive considerations. For convenience, especially in view of the stochastic generalization of the CQG theory to be later discussed in Section 3, the content is organized into the following subsections:

- Section 2.1: GLP representation of CQG theory.
- Section 2.2: Properties of GLP theory.
- Section 2.3: Construction method for the quantum-modified EFE.

### 2.1. GLP Representation of CQG Theory

For definiteness, we briefly present the theoretical setup of CQG theory in the context of the trajectory-based GLP representation. The starting point is represented by the manifestly-covariant four-scalar quantum gravity wave equation (CQG-wave equation) obtained in [37,38]. In the validity of the unitarity principle, this equation takes the form of the hyperbolic first-order Eulerian PDE:

(2) $$i\hbar \frac{d}{ds}\psi \left(s\right)=H_{R}^{\left(q\right)}\psi \left(s\right),$$

with *ℏ* being the reduced Planck constant, $\frac{d}{ds}={\left.\frac{d}{ds}\right|}_{s}+\frac{\partial }{\partial s}$ denoting the covariant *s*-derivative in Eulerian form, where the first differential operator is the customary covariant derivative evaluated at fixed *s* and the second one is a partial derivative acting on explicit proper-time dependences. In addition, $H_{R}^{\left(q\right)}$ represents a suitable self-adjoint quantum Hamiltonian operator introduced in [37]. For brevity, we omit reporting the mathematical definition of the operator $H_{R}^{\left(q\right)}$ because it is not necessary at this stage, while we prefer to focus below on the expression of the corresponding function $H^{\left(q\right)}$ that enters the quantum hydrodynamic equations that follow from (2). Furthermore, ψ(s) stands for $\psi \left(s\right)\equiv \psi \left(g,\hat{g},r,s\right)$ and denotes the four-scalar quantum wave function associated with a graviton particle, which is defined in principle for arbitrary values of the proper time *s* belonging to the time axis I≡R. Both explicit and implicit dependences on *s* are allowed, the latter entering through the four-position vector $r\equiv r^{\mu }\left(s\right)$ of the background space-time. In addition, a functional dependence on both $g=\left\{g_{\mu \nu }\right\}$ and $\hat{g}=\left\{{\hat{g}}_{\mu \nu }\right\}$ is included, where $g_{\mu \nu }$ is the quantum generalized-coordinate field, which spans the 10-dimensional real vector space $U_{g}\subseteq R^{10}$ of the same wave-function, i.e., the set on which the associated quantum probability density function $\rho \left(s\right)={\left|\psi (s)\right|}^{2}$ (quantum PDF) is prescribed, while ${\hat{g}}_{\mu \nu }$ is the background metric tensor. In this regard, it is important to recall that a crucial feature of CQG theory lies in the distinction between the quantum tensor $g_{\mu \nu }$, which identifies the continuum Lagrangian coordinates carrying the quantum physical properties of the gravitational field, and the background metric tensor ${\hat{g}}_{\mu \nu }$, which instead describes the geometry of space-time. By definition, the tensor $g_{\mu \nu }$ is such that $g^{\mu \nu }g_{\mu \nu }\neq \delta_{\mu }^{\mu }$, while identically, the normalization condition ${\hat{g}}^{\mu \nu }{\hat{g}}_{\mu \nu }=4$ applies to the classical field. Accordingly, the quantum field $g_{\mu \nu }$ is allowed to exhibit a quantum dynamical behavior, which deviates from ${\hat{g}}_{\mu \nu }$, and to acquire a non-vanishing quantum momentum $\Pi_{\mu \nu }$.

The CQG-wave Equation (2) has formal similarities with the Schroedinger equation of non-relativistic quantum mechanics. This includes also its equivalence with a corresponding set of quantum hydrodynamics equations, which can be similarly recovered upon introducing an exponential representation for the complex field ψ(s), i.e., the so-called Madelung representation:

(3) $$\psi \left(g,\hat{g},r,s\right)=\sqrt{\rho }\exp\left\{\frac{i}{\hbar }S^{\left(q\right)}\right\}.$$

Here, the quantum fluid fields $\left\{\rho ,S^{\left(q\right)}\right\}\equiv \left\{\rho \left(g,\hat{g},r,s\right),S^{\left(q\right)}\left(g,\hat{g},r,s\right)\right\}$ identify respectively the four-scalar quantum PDF and quantum phase-function. As a result, the same quantum fluid fields can be shown to satisfy the following set of PDEs, denoted as GR-quantum hydrodynamic equations (CQG-QHE) and identified with the continuity and quantum Hamilton–Jacobi equations:

(4) $$\begin{array}{ccc} \frac{d\rho }{ds}+\frac{\partial }{\partial g_{\mu \nu }}\left(\rho V_{\mu \nu }\right) & = & 0, \end{array}$$

(5) $$\begin{array}{ccc} \frac{dS^{\left(q\right)}}{ds}+H^{\left(q\right)} & = & 0, \end{array}$$

where $S^{\left(q\right)}\equiv S^{\left(q\right)}\left(g,\hat{g},r,s\right)$ is the quantum phase-function. In addition,

(6) $$V_{\mu \nu }\equiv \frac{1}{\kappa }\frac{\partial S^{\left(q\right)}}{\partial g^{\mu \nu }},$$

is the tensor “velocity” field, with κ being a dimensional constant, which is related to the graviton mass estimate given in [37]. Furthermore, $H^{\left(q\right)}$ denotes the effective quantum Hamiltonian density:

(7) $$H^{\left(q\right)}=\frac{1}{2\kappa }\frac{\partial S^{\left(q\right)}}{\partial g^{\mu \nu }}\frac{\partial S^{\left(q\right)}}{\partial g_{\mu \nu }}+V_{QM}+V_{o}.$$

Here, the case of vacuum conditions is considered, namely the absence of classical sources, so that $V_{o}$ and $V_{QM}$ identify respectively the vacuum effective potential and the quantum Bohm interaction potential [52] given by:

(8) $$V_{o}=\kappa \left(2-\frac{1}{4}g^{\mu \nu }g_{\mu \nu }\right)g^{\alpha \beta }{\hat{R}}_{\alpha \beta },$$

(9) $$V_{QM}\equiv \frac{\hbar^{2}}{8\kappa }\frac{\partial \ln\rho }{\partial g^{\mu \nu }}\frac{\partial \ln\rho }{\partial g_{\mu \nu }}-\frac{\hbar^{2}}{4\kappa }\frac{\partial^{2}\rho }{\rho \partial g_{\mu \nu }\partial g^{\mu \nu }},$$

where ${\hat{R}}_{\alpha \beta }$ is the Ricci tensor evaluated in terms of the background metric tensor ${\hat{g}}_{\alpha \beta }$.

The fundamental principles and conceptual meaning at the basis of GLP theory, as a trajectory-based formulation of CQG theory, can be found in [47,49]. This is achieved in terms of a suitable generalized Lagrangian path (GLP) representation for the quantum Lagrangian field $g_{\mu \nu }\left(s\right)$ of the form:

(10) $$g_{\mu \nu }\left(s\right)=\Delta g_{\mu \nu }+G_{\mu \nu }\left(s\right),$$

where $G_{\mu \nu }\left(s\right)$ (see the extended related discussion in [47]) denotes a suitably-prescribed reference *s*-dependent quantum field, to be associated with a suitable Lagrangian path $\left\{G_{\mu \nu }\left(s\right),s\in I\right\}$ (which can be effectively treated as deterministic if its initial value $G_{\mu \nu }\left(s_{o}\right)$ is considered deterministic). Instead, the tensor displacement field $\Delta g_{\mu \nu }$ is assumed as an arbitrary symmetric and *s*-independent stochastic tensor field with a given probability density. The stochastic character of CQG theory in this representation emerges therefore as a natural consequence of Equation (10). The meaning of Equation (10) is that for each (deterministic) Lagrangian trajectory $\left\{G_{\mu \nu }\left(s\right),s\in I\right\}$, there are infinite stochastic GLPs $\left\{g_{\mu \nu }\left(s\right),s\in I\right\}$.

The GLP approach, despite the obvious non-uniqueness feature that is intrinsic in the concept of GLP, has an important implication: it allows for the construction of an explicit analytical solution for the quantum PDF $\rho \left(s\right)$. As shown in [47], this solution is represented in terms of a distribution of the type:

(11) $$\rho \left(s\right)=\rho_{G}\exp\left\{-\int_{s_{o}}^{s}ds^{\prime }\frac{\partial V_{\nu }^{\mu }\left(s^{\prime }\right)}{\partial g_{\nu }^{\mu }\left(s^{\prime }\right)}\right\},$$

where $V_{\nu }^{\mu }\left(s\right)\equiv {\hat{g}}^{\mu \alpha }V_{\alpha \nu }\left(s\right)$ identifies the tensor velocity field (6). Furthermore, $\rho_{G}=\rho \left(\Delta g-\hat{g}\right)$ is the shifted Gaussian PDF:

(12) $$\rho_{G}\equiv \frac{1}{\pi^{5}r_{th}^{10}}\exp\left\{-\frac{{\left(\Delta g-\hat{g}\right)}^{2}}{r_{th}^{2}}\right\},$$

with $r_{th}^{2}$ being the constant dimensionless invariant semi-amplitude width of the Gaussian quantum PDF, while in short-notation, the exponent ${\left(\Delta g-\hat{g}\right)}^{2}$ stands for the four-scalar defined as ${\left(\Delta g-\hat{g}\right)}^{2}\equiv \left(\Delta g_{\mu \nu }-{\hat{g}}_{\mu \nu }\right)\left(\Delta g^{\mu \nu }-{\hat{g}}^{\mu \nu }\right)$. More precisely, $\hat{g}={\hat{g}}_{\mu \nu }$ is the background metric tensor, and $\Delta g\equiv \Delta g_{\mu \nu }$ identifies the stochastic displacement symmetric tensor field associated with each stochastic quantum trajectory and such that it has an identically vanishing covariant derivative, namely $\frac{D}{Ds}\Delta g_{\mu \nu }=0$. As proven in [47], the generalized Lagrangian path theory can be constructed in such a way that the stochastic average over Δg of quantum observables (denoted with the symbol <>) coincides with their quantum expectation value, so that in particular, the emergent gravity relationship $\langle \Delta g_{\mu \nu }\rangle ={\hat{g}}_{\mu \nu }$ holds. This is obtained by noting that, under suitable assumptions, the four-scalar $\frac{\partial V_{\nu }^{\mu }\left(s^{\prime }\right)}{\partial g_{\nu }^{\mu }\left(s^{\prime }\right)}$ does not depend explicitly on the displacement tensor Δg.

It has been proven that a quantum Hamiltonian structure analogous to that holding for the classical GR-Hamilton equations can be established also for the quantum hydrodynamic state starting from the quantum Hamilton–Jacobi Equation (5). This is represented by the set $\left\{x,H^{\left(q\right)}\right\}$, where the four-tensor canonical state $x\equiv (g_{\mu \nu },\Pi^{\mu \nu })$ is the Hamiltonian hydrodynamic state, with $\Pi^{\mu \nu }=\frac{\partial S^{\left(q\right)}}{\partial g_{\mu \nu }}$, and $H^{\left(q\right)}$ is the effective quantum Hamiltonian density defined above in Equation (7). This permits representing Equation (5) equivalently as a set of manifestly-covariant quantum Hamilton equations, which take the form of evolution equations in terms of the proper-time invariant parameter *s*. In the vacuum, these equations are written as:

(13) $$\begin{array}{ccc} \frac{d}{ds}g^{\mu \nu } & = & \frac{\Pi^{\mu \nu }}{\alpha L}, \end{array}$$

(14) $$\begin{array}{ccc} \frac{d}{ds}\Pi_{\mu \nu } & = & -\frac{\partial }{\partial g^{\mu \nu }}\left(V_{o}+V_{QM}\right), \end{array}$$

which are subject to generic initial conditions of the type $x\left(s_{o}\right)=x_{o}\equiv \left(g_{\left(o\right)}^{\mu \nu }\equiv g^{\mu \nu }\left(s_{o}\right),\Pi_{\left(o\right)\mu \nu }\equiv \Pi_{\mu \nu }\left(s_{o}\right)\right)$.

### 2.2. Properties of GLP Theory

The GLP theory represents a convenient formalism for the investigation of non-linear quantum interactions of the gravitational field with itself, namely the so-called second-quantization effects, which can have an intrinsic quantum stochastic character and be also proper-time-dependent contributions generated by the quantum wave dynamics. Effects of this type are believed to modify in turn the solution of the same background metric tensor defining the covariance property. More precisely, in such a framework, the latter tensor does not represent only a given classical tensor field, but rather it remains prescribed by the same quantum theory to retain imprints arising from its quantum dynamics. In fact, CQG theory is naturally characterized by the validity of an emergent gravity picture, to be regarded as a physical requisite of consistency, which establishes the connection between classical GR and the stochastic property of the quantum gravity theory. In detail, two types of emergent gravity were identified to hold. The first one was referred to as the first-type emergent gravity paradigm and consists of the fact that CQG theory preserves the functional form of the Einstein equations while generating self-consistent quantum corrections to the same field equations obtained without introducing the semiclassical limit. In fact, the canonical theory has permitted the explicit construction of non-perturbative quantum-modified Einstein field equations (EFE) [49,50], which can be derived from the quantum Hamiltonian equations by imposing suitable initial conditions on the hydrodynamic state $x\left(s_{o}\right)$ evaluated at initial proper-time $s_{o}$. The remarkable feature is that in both cases considered in [49,50] for different initial conditions of $x\left(s_{o}\right)$, the resulting quantum-modified EFE carry a non-vanishing stress-energy tensor associated with a quantum cosmological constant, which warrants the existence of a cosmological de Sitter solution for the corresponding quantum-modified background metric tensor $\hat{g}\equiv {\hat{g}}_{\mu \nu }$ (see also the mathematical details below in Section 2). The second feature, denoted as the second-type emergent gravity paradigm, refers instead to the possibility of prescribing the deterministic background metric tensor $\hat{g}$ by means of a suitably-defined quantum/stochastic expectation value of the quantum state. This is identified in terms of a statistical ensemble average with respect to stochastic fluctuations of the quantum gravitational field $g_{\mu \nu }$, whose quantum-wave dynamics is described by means of GLP trajectories [47]. For the sake of reference, it may be worthwhile to recall here some of the key results obtained so far by the CQG theory concerning the issue of the quantum origin of the cosmological constant. The following outcomes have been pointed out:

(1) In [49], it was proven that CQG theory yields a well-defined quantum prescription of the cosmological constant and of its physical interpretation. This is ascribed to a quantum gravity second-quantization effect due to the action of the non-linear Bohm quantum vacuum interaction of the gravitational field with itself. The existence of the Bohm potential therefore warrants the outcome of a cosmological constant term in the Einstein field equations. The result depends generally on the realization of the quantum probability density for the quantum gravitational field tensor and the related prescription of the quantum vacuum density obtained in such a framework. The emerging physical picture predicts a generally non-stationary (with respect to the proper-time parameter) quantum cosmological constant, which originates from fluctuations (i.e., gradients) of quantum vacuum gravitational energy density, rather than the vacuum energy density itself, and is consistent with the existence of quantum massive gravitons.

(2) In [50], small-amplitude quantum gravity periodic perturbations of the metric tensor, occurring in sequences of phase-shifted oscillations, were investigated for vacuum conditions, based on the Hamiltonian representation of the quantum hydrodynamic equations of CQG theory subject to non-stationary initial conditions for the canonical momenta. It was shown that for the de Sitter space–time, these perturbations give rise to non-linear modifications of the Einstein field equations in terms of a stochastically-averaged and divergence-free quantum stress-energy tensor. As a result, a quantum-driven screening effect was proven to occur, which can affect the magnitude of the cosmological constant. This means that the action of non-stationary momenta of the quantum gravitational field can decrease the content of the Bohm potential through quantum Hamiltonian dynamics and reduce in this way the absolute value of the quantum cosmological constant generated by its gradient.

(3) In [51], the consistency between the prescription of the cosmological constant by CQG theory and its role in the classical variational theory of the Einstein equations was established. In particular, the manifestly-covariant Hamiltonian structure of classical general relativity was shown to be associated with a path-integral synchronous Hamilton variational principle for the Einstein field equations, to be realized either in unconstrained or constrained forms. As a consequence, the cosmological constant was found to be identified with a Lagrange multiplier associated with the normalization constraint for the extremal metric tensor, thus identifying a four-scalar gauge function generally dependent on an invariant proper-time parameter *s*, consistent with the prediction of the cosmological constant based on the theory of manifestly-covariant quantum gravity.

### 2.3. Construction Method for the Quantum-Modified EFE

As pointed out in [38], Equations (13) and (14) can be used to construct a suitable stationary solution of the canonical state, i.e., for which identically:

(15) $$\frac{d}{ds}g^{\mu \nu }=\frac{d}{ds}\Pi_{\mu \nu }=0.$$

This requirement is actually sufficient in the framework of the classical Hamiltonian theory of GR developed in [37] to determine the classical EFE, which in the present notation and under the assumption of vacuum conditions are written as:

(16) $${\hat{R}}_{\mu \nu }-\frac{1}{2}\hat{R}{\hat{g}}_{\mu \nu }=0.$$

However, the rhs of Equation (14) still generally depends on the arbitrary stochastic tensor field $\Delta g_{\mu \nu }$. The same quantum Hamilton Equations (13) and (14) were proven in [49,50] to generate a corresponding quantum-modified EFE. This can be generally written in tensor notation as:

(17) $${\hat{R}}_{\mu \nu }-\frac{1}{2}\hat{R}{\hat{g}}_{\mu \nu }=T_{\mu \nu }^{\left(q\right)},$$

with $T_{\mu \nu }^{\left(q\right)}$ denoting a suitable quantum stress-energy tensor prescribed by CQG theory and generally dependent on proper-time. Its expression follows from the gradient of the Bohm potential $V_{QM}$ in Equation (14), from the actual solution for the quantum PDF $\rho \left(s\right)$, and from possible contributions due to non-vanishing quantum momenta. Let us now explain how the analytical form of $T_{\mu \nu }^{\left(q\right)}$ entering the quantum-modified EFE is actually achieved. This is obtained by following a two-step procedure:

(1) First, taking the limit condition:

(18) $$\Delta g_{\mu \nu }\to 0$$

in Equations (13) and (14) when the representation (10) is set. This is referred to as the deterministic GLP-limit condition. Its physical meaning is intuitive: it amounts to imposing that the stochastic GLP quantum trajectories $\left\{g_{\mu \nu }\left(s\right),s\in I\right\}$, which drive the quantum wave-function, collapse on the single deterministic Lagrangian path (LP) Bohmian trajectory $\left\{G_{\mu \nu }\left(s\right),s\in I\right\}$ indicated above. Hence, from the physical standpoint, with such a requirement, the stochastic effect due to the GLP trajectories is dropped. At the same time, the customary deterministic character of the Einstein field equations is recovered, so that the same equations actually depend only on the background space-time metric tensor ${\hat{g}}_{\mu \nu }$. Nevertheless, non-vanishing quantum gravity contributions can still appear. For completeness, we mention here that the limit condition (18) is conceptually different from taking the stochastic average over $\Delta g_{\mu \nu }$ of the resulting quantum-modified EFE. In the former case in fact, the system becomes intrinsically deterministic, so that any stochastic contribution due to $\Delta g_{\mu \nu }$ is effectively set to zero, while in the latter one, only the average of the stochastic system is obtained.

(2) Second, the initial state $x\left(s_{o}\right)$ for the initial-value problem associated with the quantum Hamilton Equations (13) and (14) is prescribed. Imposing vanishing initial conditions, namely such that:

(19) $$x\left(s_{o}\right)=\left(g_{\left(o\right)}^{\mu \nu }\equiv g^{\mu \nu }\left(s_{o}\right),\Pi_{\left(o\right)\mu \nu }\equiv 0\right),$$

yields the quantum-modified Einstein field equations determined in [49]. This means that the initial quantum tensor $g^{\mu \nu }$ coincides with the background one, and its corresponding momentum (i.e., its covariant derivative) is identically vanishing. Notice that in the GLP representation given by Equation (10), this means that in the validity of (18), one obtains:

(20) $$g_{\mu \nu }\left(s\right)=\Delta g_{\mu \nu }+G_{\mu \nu }\left(s\right)=G_{\mu \nu }\left(s\right).$$

Hence, requiring

(21) $$G_{\mu \nu }\left(s\right)={\hat{g}}_{\mu \nu }\left(s\right)$$

to be equivalent to letting:

(22) $$g_{\mu \nu }\left(s\right)={\hat{g}}_{\mu \nu }\left(s\right).$$

The order in which Assumptions (1) and (2) are taken is irrelevant, which means that Condition (2) can be equivalently replaced requiring Equation (22). It follows that the tensor $T_{\mu \nu }^{\left(q\right)}$ is uniquely determined in terms of the Bohm quantum potential, and it actually coincides with the tensor $B_{\mu \nu }$ reported in [49], namely in such a case $T_{\mu \nu }^{\left(q\right)}=B_{\mu \nu }$. One finds that this tensor is a function of the quantum-gravitational cosmological constant $\Lambda_{CQG}\left(s\right)$ determined in [49], namely of the form $B_{\mu \nu }=\Lambda_{CQG}\left(s\right){\hat{g}}_{\mu \nu }$. Here, $\Lambda_{CQG}\left(s\right)$ arises due to the Bohm quantum vacuum interaction among massive gravitons and is generally a function of proper-time with the initial value at $s=s_{o}$ given by $\Lambda_{CQG}\left(s_{o}\right)$, where:

(23) $$\Lambda_{CQG}\left(s_{o}\right)=\frac{\hbar^{2}}{\kappa^{2}}\frac{1}{r_{th}^{4}},$$

and *ℏ* is the reduced Planck constant.

For completeness, we mention, however, that in principle, also non-equilibrium initial conditions of the type:

(24) $$x\left(s_{o}\right)=\left(g_{\left(o\right)}^{\mu \nu }\equiv g^{\mu \nu }\left(s_{o}\right),\Pi_{\left(o\right)\mu \nu }\equiv \delta \pi_{\mu \nu }\left(s_{o}\right)\right),$$

with $\delta \pi_{\mu \nu }\left(s_{o}\right)\neq 0$ being infinitesimal, can be adopted. This initial condition provides the momentum quantum-modified EFE discussed in [50] leading to the identification of the quantum-screening mechanism for the absolute value of the cosmological constant. In this case, the tensor $T_{\mu \nu }^{\left(q\right)}$ is shown to carry both the contribution from the Bohm potential and proper-time averaged quantum contributions of the ensemble of perturbations generated by fluctuations of quantum gravitational field and conjugate momentum. Remarkably, also in this case, the same stress-energy tensor can be expressed in terms of a screened quantum-gravitational cosmological constant $\Lambda_{S}=K\Lambda_{CQG}\left(s_{o}\right)$, where K<1 is a suitable numerical correction factor estimated in [50]. More precisely, one finds that $T_{\mu \nu }^{\left(q\right)}=\Lambda_{S}{\hat{g}}_{\mu \nu }$, and this expression generalizes the one of $B_{\mu \nu }$ by means of the inclusion of screening factor *K*.

A remarkable consequence of the initial conditions indicated by Equation (19) and Equation (24) is that both admit a de Sitter solution for the metric tensor ${\hat{g}}_{\mu \nu }$, subject to corresponding prescribed quantum corrections carried by the cosmological constant term. In particular, upon introducing the four-scalar function $B\equiv \left(1-\frac{r^{2}}{A^{2}}\right)$, the background metric tensor in spherical coordinates (ct,r,ϑ,φ) can be written as ${\hat{g}}_{\mu \nu }=diag\left\{B,B^{-1},r^{2},r^{2}\sin^{2}\vartheta \right\}$, so that the corresponding Riemann distance takes the form $ds^{2}=Bc^{2}dt^{2}-B^{-1}dr^{2}+r^{2}d\Omega^{2}$. Here, the parameter *A* is related to the “radius” of the de Sitter space-time, which in the framework of the quantum modifications of EFE considered above depends on either $\Lambda =\Lambda_{CQG}$ or $\Lambda =\Lambda_{S}$, respectively in the two cases discussed above, by means of the prescription $A=\sqrt{\frac{3}{\Lambda }}$. It must be stressed that the solutions obtained so far include quantum corrections to space-time ${\hat{g}}_{\mu \nu }$ holding under the deterministic condition (18), so that in previous treatments, stochastic contributions to EFE due to the stochastic variable $\Delta g_{\mu \nu }$ and predicted by GLP theory were ruled out in this way. For the same reason, the de Sitter solution constructed above yields a deterministic representation for the metric tensor ${\hat{g}}_{\mu \nu }$ and the physical properties of space-time associated with its cosmological meaning.

## 3. Stochastic Quantum Gravity

We now carry out explicitly the stochastic generalization of CQG theory. The task is reached by extending the CQG theory and its GLP representation given in [47] in such a way to include a new kind of physically-admissible stochastic effects. We intend to show that such effects:

- are actually consistent with the dynamics of the quantum gravitational field;
- have an intrinsic indeterminacy character, because the new stochastically-modified PDF introduced here does not affect the quantum Hamilton Equations (13) and (14);
- give rise to non-trivial modifications of the corresponding quantum-modified EFE and background field tensor.

We stress that by construction, GLP theory is intrinsically stochastic. Such a feature arises thanks to the stochastic displacement tensor $\Delta g_{\mu \nu }$, which determines also the statistical distribution of Lagrangian trajectories around the deterministic Bohmian path and also the quantum PDF $\rho \left(s\right)$. However, the procedure adopted for the construction of the modified EFE (recalled above) requires ultimately dropping altogether this type of stochastic effect.

Nevertheless, it is possible to prove that a more general solution for the quantum gravity PDF can be reached, which carries an additional stochastic contribution expressed by a four-scalar stochastic variable denoted here by the symbol α and having a prescribed probability density function. The new stochastic effect is actually independent of the GLP stochastic displacement tensor $\Delta g_{\mu \nu }$. Its physical meaning is therefore unrelated to GLP theory, but rather to the nature of stochastic physical observables arising in quantum gravity (see the discussion below). As a notable feature, the α-stochastic parameter does not change the GLP stochastic trajectories determined in [47], so that the same GLP theory previously constructed still applies. It follows that the stochastic contribution of parameter α preserves the probabilistic character of the new solution for the quantum gravity PDF, denoted by $\rho_{\alpha }\left(s\right)$, as well as its unitarity principle.

In detail, let us assume that $\alpha \in \left[\alpha_{0},\alpha_{1}\right]$,with $\alpha_{0}<\alpha_{1}$, is a four-scalar stochastic parameter independent of $g_{\mu \nu }$ and *s*. Then, the quantum PDF (11) can be formally extended to include a dependence on α, while still retaining its Gaussian character. The solution to the continuity equation can always be taken in the form $\rho_{\alpha }\left(s\right)\equiv \rho \left(\Delta g\left(\alpha \right),\alpha ,\hat{g}\left(s,\alpha \right)\right)$, where:

(25) $$\rho_{\alpha }\left(s\right)=\rho_{G}\left(s,\alpha \right)\exp\left\{-\int_{s_{o}}^{s}ds^{\prime }\frac{\partial V_{\nu }^{\mu }\left(s^{\prime }\right)}{\partial g_{\nu }^{\mu }\left(s^{\prime }\right)}\right\},$$

$V_{\nu }^{\mu }\left(s\right)$ is the tensor velocity field (6) assumed independent of α and $\rho_{G}\left(s,\alpha \right)$ is the shifted Gaussian PDF (denoted as α-Gaussian PDF), which is now expressed in terms of the stochastic tensor displacement Δg(α) (see Equation (1)):

(26) $$\rho_{G}\left(s,\alpha \right)=K\exp\left\{-\frac{{\left(\Delta g\left(\alpha \right)-\hat{g}\left(s,\alpha \right)\right)}^{2}}{r_{th}^{2}}\right\}g\left(\alpha ,\varepsilon \right),$$

with g(α,ε) denoting an in principle arbitrary four-scalar stochastic PDF. As an example, g(α,ε) can be identified with a Gaussian PDF:

(27) $$g\left(\alpha ,\varepsilon \right)=N\exp\left\{-\frac{\alpha^{2}}{\varepsilon^{2}}\right\},$$

with ordinarily finite support, so that $\alpha \in \left[\alpha_{0},\alpha_{1}\right]\subset R$ (this assumption however may on occasion not be required for numerical estimates) while *N* is a normalization constant and ε is a suitably small dimensionless factor to be assumed ε≪1 (see below). In Equation (26), *K* is therefore the normalization factor:

(28) $$K={\left[\int_{U_{g}}d\left(\Delta g\right)\int_{\alpha_{0}}^{\alpha_{1}}d\alpha \exp\left\{-\frac{{\left(\Delta g\left(\alpha \right)-\hat{g}\left(s,\alpha \right)\right)}^{2}}{r_{th}^{2}}\right\}g\left(\alpha ,\varepsilon \right)\right]}^{-1}.$$

In Equation (26), the tensors $\Delta g\left(\alpha \right)\equiv \Delta g_{\mu \nu }\left(\alpha \right)$ and $\hat{g}\left(s,\alpha \right)\equiv {\hat{g}}_{\mu \nu }\left(s,\alpha \right)$ identify the generalized displacement tensor $\Delta g_{\mu \nu }$ of GLP theory and the background metric tensor, both now admitted to generally depend on α itself, while $\hat{g}$ can also depend on the proper-time *s*. Similarly, in the same equation, the exponent ${\left(\Delta g\left(\alpha \right)-\hat{g}\left(s,\alpha \right)\right)}^{2}$ stands for the four-scalar defined as ${\left(\Delta g\left(\alpha \right)-\hat{g}\left(s,\alpha \right)\right)}^{2}\equiv \left(\Delta g_{\mu \nu }\left(\alpha \right)-{\hat{g}}_{\mu \nu }\left(s,\alpha \right)\right)\left(\Delta g^{\mu \nu }\left(\alpha \right)-{\hat{g}}^{\mu \nu }\left(s,\alpha \right)\right)$. As proven explicitly below, the expression for $\Delta g_{\mu \nu }\left(\alpha \right)$ can be realized so that again, the condition $\frac{D}{Ds}\Delta g_{\mu \nu }\left(\alpha \right)=0$.

The solution $\rho_{\alpha }\left(s\right)$ in Equation (26) represents a probability density since by construction:

(29) $$\langle 1\rangle =\int_{U_{g}}d\left(\Delta g\right)\int_{\alpha_{0}}^{\alpha_{1}}d\alpha \rho_{\alpha }\left(s\right)=1,$$

which means that the quantum continuity Equation (4) preserves quantum unitarity. In addition, it preserves the second-type emergent gravity feature of CQG theory (see [47]), namely one has that again:

(30) $$\langle \Delta g_{\mu \nu }\left(\alpha \right)\rangle =\int_{U_{g}}d\left(\Delta g\right)\int_{\alpha_{0}}^{\alpha_{1}}d\alpha \left[\Delta g_{\mu \nu }\left(\alpha \right)\rho_{\alpha }\left(s\right)\right]={\hat{g}}_{\mu \nu }.$$

We notice that in the limit α→0, one recovers the previous GLP theory summarized in Section 2. In addition, the distribution of α is a Gaussian centered around zero and with half-width ε, so that its stochastic average is identically null, namely:

(31) $$\langle \alpha \rangle =\int_{U_{g}}d\left(\Delta g\right)\int_{\alpha_{0}}^{\alpha_{1}}d\alpha \left[\alpha \rho_{\alpha }\left(s\right)\right]=0.$$

In this stochastic model, the contribution carried by α leaves invariant the GLP theory developed earlier. The form of the solution in Equation (25) therefore provides a consistent generalization for the representation of the quantum gravity PDF, which follows from the indeterminacy property of the solution of the continuity equation with respect to the dependence on the stochastic parameter α and the validity of the GLP formulation of CQG theory. In fact, regarding the replacement introduced here in Equation (25) with respect to [47], namely:

(32) 1→g(α,ε),

and consequently, $\rho_{G}\left(s,\alpha =0\right)\to \rho_{G}\left(s,\alpha \right)$, it must be remarked that the specific form of the stochastic PDF g(α,ε) does not enter explicitly, as previously for $\rho_{G}\left(s,\alpha =0\right)$, in the quantum Hamilton Equations (13) and (14).

For completeness we notice that two possible realizations of Equation (25) could be distinguished, depending on the functional form of the half-width parameter ε, namely respectively:

**Case** **1.** *In the first case, one can set ε=const.≪1 everywhere. This choice preserves the validity of the quantum continuity equation and is therefore consistent with the unitary principle, and correspondingly of a quantum-unitary formulation of CQG theory. Equation (26) preserves its validity as a solution of the continuity equation, since the dependence on α represents a property of the indeterminacy of the same PDF that generalizes the solution obtained earlier, while leaving unaltered the theoretical framework of the GLP formalism. In such a framework, the stochastic PDF (25) holds in the whole space-time.*

**Case** **2.** *For the sake of completeness, however, we mention that a further more general realization can in principle be reached. This is obtained by assuming a proper-time dependence for the half-width parameter ε, namely of the type ε=ε(r(s)), with ε(r(s)) being an arbitrary, but suitably smooth function. Such a choice preserves quantum unitarity by construction, but, following the guidelines of [32], requires replacing the quantum continuity Equation (4) with an inhomogeneous one of the form:*

(33) $$\frac{d\rho_{q}}{ds}+\frac{\partial }{\partial g_{\mu \nu }}\left(\rho_{q}V_{\mu \nu }\right)=Q_{L}^{\left(q\right)},$$

*with $Q_{L}^{\left(q\right)}$ denoting the quantum “source” term:*

(34) $$Q_{L}^{\left(q\right)}=\rho_{q}\left(\Delta g\left(\alpha \right),\alpha ,\hat{g}\left(s,\alpha \right)\right)\frac{2\alpha^{2}}{\varepsilon^{2}\left(r\left(s\right)\right)}\frac{d\ln\varepsilon (r(s))}{ds}.$$

Notice that, independent of the prescription of the function ε(r) and due to the normalization (29), $Q_{L}^{\left(q\right)}$ gives nevertheless a vanishing contribution upon the integration indicated in the same normalization. Therefore, unlike [32], this is not actually a net source or sink term. Therefore, its physical meaning remains problematic. Nevertheless, if $Q_{L}^{\left(q\right)}$ is considered prescribed, the previous equation can also be used in principle to determine the profile of ε in terms of the quantum term $Q_{L}^{\left(q\right)}=Q_{L}^{\left(q\right)}\left(s\right)$. Hence, its inclusion could be useful for the search of stochastic effects when a combination of loss/capture mechanisms is present in a domain that is located suitably close to the de Sitter event horizon. Thus, if $r_{EH}^{\left(1\right)}$ and $r_{EH}^{\left(2\right)}$ are two different space-time positions identified with suitable “internal” and “external” radii with respect to the event horizon radius $r_{EH}$ of the de Sitter solution, so that by construction $r_{EH}^{\left(1\right)}<r_{EH}<r_{EH}^{\left(2\right)}$, a possible choice is to assume that:

(35) $$\varepsilon^{2}\left(r(s)\right)=\left\{\begin{array}{c} \varepsilon^{2}\left(r(s)\right)>0\;\mathrm{for}\;r_{EH}^{\left(1\right)}<r<r_{EH}^{\left(2\right)}\\ \varepsilon_{o}^{2}=const.\;\mathrm{for}\;r<r_{EH}^{\left(1\right)} \end{array}\right\},$$

where the limits apply:

(36) $$\lim_{r\to r_{EH}^{\left(1\right)}}\frac{1}{\varepsilon^{2}\left(r(s)\right)}\frac{d\ln\varepsilon (r(s))}{ds}=\lim_{r\to r_{EH}^{\left(2\right)}}\frac{1}{\varepsilon^{2}\left(r\left(s\right)\right)}\frac{d\ln\varepsilon (r(s))}{ds}=0.$$

It follows that, in this realization of the quantum PDF, the set $I_{r}\equiv \left\{\left.r\right|r_{EH}^{\left(1\right)}<r<r_{EH}^{\left(2\right)}\right\}$ denotes the stochastic set surrounding the de Sitter event horizon where the quantum source term $Q_{L}^{\left(q\right)}$ is non-zero. Furthermore, if additionally, $\varepsilon_{o}^{2}$ is taken to be an infinitesimal dimensionless parameter such that:

(37) $$\varepsilon_{o}^{2}\ll r_{th}^{2},$$

then, as a consequence, outside the subset $I_{r}$, the shifted α-Gaussian quantum PDF becomes:

(38) $$\rho_{G}\left(s,\alpha \right)=K\exp\left\{-\frac{{\left(\Delta g\left(\alpha \right)-\hat{g}\left(s,\alpha \right)\right)}^{2}}{r_{th}^{2}}-\frac{\alpha^{2}}{\varepsilon_{o}^{2}}\right\}≅K_{o}\exp\left\{-\frac{{\left(\Delta g-\hat{g}\left(s\right)\right)}^{2}}{r_{th}^{2}}\right\}\delta \left(\alpha \right),$$

so that effectively outside the same set, the contribution of the stochastic parameter α can be safely ignored and $K_{o}$ denotes the normalization factor in the limit α→0. Accordingly, this model provides a convenient solution for the study of stochastic effects near the de Sitter event horizon where the contribution due to the stochastic parameter is non-vanishing and can be associated with the quantum sink $Q_{L}^{\left(q\right)}$. In this regard, however, we notice that the precise realization of $Q_{L}^{\left(q\right)}$ must be physically-motivated, as well as the physical mechanisms responsible for its occurrence in the stochastic set $I_{r}$ and its vanishing limit in the remaining region.

Based on these considerations, in the following, we shall adopt the framework corresponding to Case 1, so that we shall assume ε=const. everywhere. The investigation of the physical meaning of the solution corresponding to Case 2 is left to future studies.

## 4. Stochastic Quantum-Modified Einstein Equations

Based on the extended solution for the quantum gravity PDF obtained in Section 3, which includes the novel contribution due to the quantum gravity stochastic factor α predicted by CQG theory, in this section, we consider the consequences of such a dependence on the quantum-modified Einstein equations and on the validity of a cosmological de Sitter solution for the space-time metric tensor. For convenience and without loss of generality, to reach the goal, we adopt the setting provided by Equation (19) reported above for the choice of “equilibrium” initial conditions of the quantum state. This allows us to single out the new α-stochastic contribution and gain insight into its role in the Einstein equations. The extension of the solution to the case of “non-equilibrium” initial state will be then discussed at the end of the section. For the same reason, we exclude here the possible occurrence of external source fields other than the gravitational one. In detail, in such a setting and without performing the semiclassical limit ℏ→0, Equations (13) and (14) subject to the initial condition (19) reduce to the single equation:

(39) $${\left.\frac{\partial }{\partial g^{\mu \nu }}\left(V_{o}+V_{QM}\right)\right|}_{g_{\mu \nu }={\hat{g}}_{\mu \nu }}=0,$$

which contains the information by the Hamiltonian potential. Then, invoking the definitions (8) and (9) for the potential terms $V_{o}$ and $V_{QM}$, respectively, it is straightforward to perform the differentiation with respect to $g^{\mu \nu }$ and evaluate the result for $g_{\mu \nu }={\hat{g}}_{\mu \nu }$ (which expresses a condition determined by imposing the validity of Equation (19)). This yields the following form for the resulting Einstein field equations:

(40) $${\hat{R}}_{\mu \nu }-\frac{1}{2}\hat{R}{\hat{g}}_{\mu \nu }\left(s,\alpha \right)=B_{\mu \nu }^{\left(\alpha \right)}.$$

Here, the α-stochastic quantum contribution arising from the Bohm potential is carried by the symmetric tensor $B_{\mu \nu }^{\left(\alpha \right)}$, to be distinguished from the tensor $B_{\mu \nu }$ obtained in [49] because of the new dependence on the stochastic parameter α in the quantum gravity PDF. In the following, Equation (40) will be referred to as the α-stochastic quantum-modified EFE. In particular, the stochastic tensor $B_{\mu \nu }^{\left(\alpha \right)}$ is obtained as:

(41) $$B_{\mu \nu }^{\left(\alpha \right)}\equiv {\left.-\frac{1}{\kappa }\frac{\partial }{\partial g^{\mu \nu }}V_{QM}\right|}_{g_{\mu \nu }={\hat{g}}_{\mu \nu }}=\frac{\hbar^{2}}{\kappa^{2}}\frac{f(s)}{r_{th}^{4}}\left(\Delta g_{\mu \nu }\left(\alpha \right)-{\hat{g}}_{\mu \nu }\left(s,\alpha \right)\right),$$

where f(s) is a suitably-prescribed four-scalar function depending on proper-time *s* and satisfying a given differential equation determined in [49]. Its value for the initial condition $s=s_{o}$ is such that $f(s_{o})=1$. This function carries the proper-time dependence of the quantum gravity cosmological constant, which is in this way transferred to the metric tensor ${\hat{g}}_{\mu \nu }$. Recalling the notation of Equation (23) and defining:

(42) $$\Lambda_{CQG}\left(s\right)=\frac{\hbar^{2}}{\kappa^{2}}\frac{f(s)}{r_{th}^{4}}=\Lambda_{CQG}\left(s_{o}\right)f\left(s\right),$$

we can then write Equation (40) in the explicit form:

(43) $${\hat{R}}_{\mu \nu }-\frac{1}{2}\hat{R}{\hat{g}}_{\mu \nu }\left(s,\alpha \right)=-\Lambda_{CQG}\left(s\right){\hat{g}}_{\mu \nu }\left(s,\alpha \right)+\Lambda_{CQG}\left(s\right)\Delta g_{\mu \nu }\left(\alpha \right),$$

where on the rhs, the first term identifies the quantum gravity cosmological constant term, while the second tensor carries the stochastic contributions due to $\Delta g_{\mu \nu }$ and α.

In order to express in a convenient way the tensor $\Delta g_{\mu \nu }\left(\alpha \right)$ and realize it consistently with the constraints set by the underlying quantum GLP theory, it is instructive to make a comparison with the case of EFE having a perfect fluid as a source term. In fact, we notice that in Equation (43), the role of the symmetric tensor contribution $\Delta g_{\mu \nu }\left(\alpha \right)$ can be formally associated with a symmetric stress-energy tensor $T_{\mu \nu }$, which is due to external fields and is the source of curvature. More precisely, in the case of a perfect-fluid, the stress-energy tensor is customarily written as:

(44) $$T_{\mu \nu }=\left(\epsilon +\frac{P}{c^{2}}\right)U_{\mu }U_{\nu }+P{\hat{g}}_{\mu \nu },$$

where ϵ is the energy density and *P* is the isotropic pressure. The stress-energy tensor of a perfect fluid contributes in two ways: one is the tensor contribution proportional to the fluid four-velocities $U_{\mu }U_{\nu }$ and the other one is the scalar contribution due to the pressure *P* only and having tensorial directions along the background metric tensor. We can similarly introduce a decomposition of this type for the stochastic tensor $\Delta g_{\mu \nu }\left(\alpha \right)$, thus letting:

(45) $$\Delta g_{\mu \nu }\left(\alpha \right)=\Delta g_{\mu \nu }+\alpha {\hat{g}}_{\mu \nu }\left(s,\alpha \right).$$

In particular, this representation amounts to assuming a polynomial dependence of $\Delta g_{\mu \nu }\left(\alpha \right)$ on α and ${\hat{g}}_{\mu \nu }$, retaining in the previous equation only the first-order term (i.e., linear term). This representation is consistent with the constraint that in the limit α→0, the stochastic contribution of α vanishes, and one recovers that in the same limit, $\Delta g_{\mu \nu }\left(\alpha \right)\to \Delta g_{\mu \nu }$. Comparison between Equation (44) and Equation (45) shows that $\Delta g_{\mu \nu }$, which is associated with the stochastic GLP trajectories, behaves like the tensorial part of $T_{\mu \nu }$ along the fluid Lagrangian trajectories, while the term proportional to α plays the role analogous to a stochastic pressure.

Based on this representation, we can further elaborate the tensor Equation (43) by adopting the same procedure established in Section 2 for the derivation of quantum-modified EFE. In particular, by virtue of the representation (45), we proceed with the following additional steps:

(1) Deterministic LP-limit: In Equation (43), the deterministic limit $\Delta g_{\mu \nu }\to 0$ is taken, corresponding to the collapse of the stochastic quantum GLP trajectories on the unique LP trajectory.

(2) Stochastic α-contribution: In Equation (43), the four-scalar “pressure” contribution $\alpha {\hat{g}}_{\mu \nu }\left(s\right)$ is independent of $\Delta g_{\mu \nu }$ (see the discussion in Section 3), and it does not vanish when the LP-limit is imposed. This type of stochastic quantum gravity effect is retained in the field equations.

Hence, one finally obtains the following representation for the α-stochastic quantum-modified EFE:

(46) $${\hat{R}}_{\mu \nu }-\frac{1}{2}\hat{R}{\hat{g}}_{\mu \nu }\left(s,\alpha \right)=-\Lambda_{CQG}\left(s\right)\left(1-\alpha \right){\hat{g}}_{\mu \nu }\left(s,\alpha \right),$$

which exhibits the remarkable feature of being stochastic in terms of the explicit contribution of the four-scalar parameter α. The field Equation (46) therefore determines a stochastic space-time metric tensor for which ensemble averages over α should be computed.

The virtue of the representations (45) and (46) is that the linear dependence on α remains proportional to the CQG expression of the cosmological constant and to the space-time metric tensor. It follows that necessarily, the stochastic Equation (46) admits a corresponding stochastic de Sitter solution depending on the cosmological constant $\Lambda_{CQG}\left(s\right)$ and the parameter α itself. Thus, introducing the notation ${\hat{g}}_{\mu \nu }=diag\left\{B_{\alpha },B_{\alpha }^{-1},r^{2},r^{2}\sin^{2}\vartheta \right\}$ for the background metric tensor in spherical coordinates (ct,r,ϑ,φ) and denoting hereafter for brevity $\Lambda \equiv \Lambda_{CQG}\left(s\right)$, the line element in such a case is written as:

(47) $$ds^{2}=\left(1-\frac{r^{2}}{A_{\alpha }^{2}}\right)c^{2}dt^{2}-\frac{1}{\left(1-\frac{r^{2}}{A_{\alpha }^{2}}\right)}dr^{2}+r^{2}d\Omega^{2},$$

where the parameter $A_{\alpha }$ carries the stochastic contribution. It is straightforward to see that the contribution due to the quantum gravity stochastic parameter α makes the de Sitter event horizon stochastic as well. In fact, in the absence of the stochastic effect, the location of the de Sitter event horizon is fixed as a deterministic spherical surface at $r_{EH}=\sqrt{\frac{3}{\Lambda }}$. In this case instead, the radius of the event horizon is by analogy formally given by $r_{\alpha -EH}=A_{\alpha }$, where:

(48) $$r_{\alpha -EH}=\sqrt{\frac{3}{\Lambda \left(1-\alpha \right)}},$$

which however is no longer fixed as a deterministic surface because of the presence of the stochastic parameter α.

Given the Gaussian representation for the probability density of α, from the physical point of view, the condition ε≪1 means that the same distribution is very peaked and with a narrow half-width. Hence, one can reasonably assume that in actual fact, α varies randomly in the range $∼\left[-\varepsilon ,+\varepsilon \right]$. This implies that the argument of the square-root in Equation (48) remains always positive, with the factor $\left(1-\alpha \right)∼O\left(1\right)$. The behavior of α implies the notable consequence that, similarly, the de Sitter event horizon acquires a width, to be denoted as a stochastic quantum belt, so that for an order-of-magnitude estimate, we can write $r_{\alpha -EH}\in \left[r_{\alpha -EH}^{\min},r_{\alpha -EH}^{\max}\right]$. The evaluation of the extrema of such an interval can be estimated from Equation (48) by expanding the square-root. Thus, assuming α∼ε≪1, to leading-order in ε, one finds respectively:

(49) $$\begin{array}{ccc} r_{\alpha -EH}^{\min} & = & \sqrt{\frac{3}{\Lambda \left(1+|\varepsilon |\right)}}=\sqrt{\frac{3}{\Lambda }}{\left(1+|\varepsilon |\right)}^{-\frac{1}{2}}≃r_{EH}\left(1-\frac{1}{2}\left|\varepsilon \right|\right), \end{array}$$

(50) $$\begin{array}{ccc} r_{\alpha -EH}^{\max} & = & \sqrt{\frac{3}{\Lambda \left(1-|\varepsilon |\right)}}=\sqrt{\frac{3}{\Lambda }}{\left(1-|\varepsilon |\right)}^{-\frac{1}{2}}≃r_{EH}\left(1+\frac{1}{2}\left|\varepsilon \right|\right). \end{array}$$

Hence, the width of the stochastic event horizon becomes in the same approximation $\Delta r_{\alpha -EH}=r_{\alpha -EH}^{\max}-r_{\alpha -EH}^{\min}=r_{EH}\left|\varepsilon \right|$. The main implication is that the existence of a stochastic quantum belt for the event horizon with a finite width generates a region of space-time where a tunneling effect of particles/fields (including gravitons) is in principle possible, since according to this model the same event horizon does not represent anymore a classical deterministic barrier at fixed radius, i.e., a zero-measure surface separating the inner and outer domains of space-time. This calculation provides an order-of-magnitude estimate of the spatial width of the stochastic event horizon where the quantum PDF is expressed by Equation (26) and can be associated with non-vanishing particle-tunneling effects. In the same linear approximation, it then follows that the average value of the de Sitter event horizon is estimated by:

(51) $$r_{\alpha -EH}≅r_{\alpha -EH}^{\min}+\frac{r_{\alpha -EH}^{\max}-r_{\alpha -EH}^{\min}}{2}=r_{EH},$$

since in the linear approximation, positive and negative stochastic contributions to $r_{EH}$ contribute equally and cancel out. Hence, we can write that $r_{\alpha -EH}=r_{EH}+O\left(\varepsilon^{2}\right)$. However, as shown below, a more detailed calculation of the averaged radius requires the stochastic average of $r_{\alpha -EH}$ given by Equation (48) over the stochastic quantum PDF $\rho_{\alpha }\left(s\right)$ in order to evaluate analytically the $O\left(\varepsilon^{2}\right)$-corrections. In addition, we notice that although the stochastic effects due to the inclusion of α and contained in the metric tensor affect the whole space-time, because of the form of the de Sitter solution and the shape of the Gaussian PDF, for the smallness value assumed for the half-width ε, they become relevant only close to the event horizon where the ratio $\frac{r^{2}}{A_{\alpha }^{2}}∼O\left(1\right)$ , while they are accordingly negligible elsewhere. We stress that these considerations remain valid under the assumption that ε≪1 is an infinitesimal parameter. In fact, if $\varepsilon ∼O\left(1\right)$, then the solution (26) for the quantum PDF would still retain its mathematical validity, but with a different physical meaning. The case $\varepsilon ∼O\left(1\right)$ would correspond to having dominant quantum stochastic effects in the whole space-time, and therefore no longer significant at the event horizon only. This realization however is not investigated in the present work.

As a concluding remark of this section, we must comment on the relationship between the α-stochastic effect and the screening effect of the cosmological constant described in [50]. In this regard, we notice that, given the validity of Equation (46), the stochastic parameter α enters the same equations as a correction coefficient to the CQG cosmological constant. If the screening effect is taken into consideration by the prescription of a non-equilibrium initial canonical state $x_{o}$ in the quantum Hamilton equations, its contribution determines a lowering of the magnitude of the same cosmological constant by a screening factor *K*. Therefore, the same screening effect remains preserved also when the stochastic parameter α is included, as the two contributions are independent and operate separately on the final representation of the quantum-modified EFE. Therefore, the screening effect does not change the conclusions on the stochastic event horizon, but, if included, it can change the value of $r_{EH}$ around which the stochastic quantum width arises.

## 5. Stochastic Averages of Physical Observables in de Sitter Space-Time

In this section, we consider the calculation of stochastic averages over the PDF $\rho_{\alpha }\left(s\right)$ for physical observables of interest in the de Sitter space-time, which depend on the stochastic parameter α. By definition, given an observable quantity *y*, its stochastic α-average is denoted with $\langle y\rangle$ and is defined as:

(52) $$\langle y\rangle \equiv \int_{U_{g}}d\left(\Delta g\right)\int_{\alpha_{0}}^{\alpha_{1}}d\alpha \left[y\rho_{\alpha }\left(s\right)\right]≅\int_{U_{g}}d\left(\Delta g\right)\int_{-\infty }^{\infty }d\alpha \left[y\rho_{\alpha }\left(s\right)\right],$$

where it is assumed that the PDF $\rho_{\alpha }\left(s\right)$ is normalized to one when both integrations over Δg and α are computed. Notice that in the previous equation, specifically in reference to the prescription of the stochastic PDF Equation (27), the support of the stochastic parameter α has been identified for the convenience of calculation with R. We consider here in particular the following observable quantities:

(1) The quantum cosmological constant.
(2) The de Sitter radius.
(3) The Hawking temperature.

(1) The quantum cosmological constant: 

Given the validity of the α-stochastic quantum-modified EFE (46), it is possible to identify the contribution of the effective stochastic cosmological constant $\Lambda \left(\alpha \right)$, which is provided by the absolute value:

(53) $$\Lambda \left(\alpha \right)=\Lambda_{CQG}\left(s\right)\left(1-\alpha \right).$$

The expression of $\Lambda \left(\alpha \right)$ does not depend on the construction of the stochastic tensor Δg, but only on α. Hence, the stochastic average over the Gaussian PDF $\rho_{\alpha }\left(s\right)$ yields:

(54) $$\langle \Lambda \left(\alpha \right)\rangle \equiv \int_{U_{g}}d\left(\Delta g\right)\int_{-\infty }^{+\infty }d\alpha \left[\Lambda \left(\alpha \right)\rho_{\alpha }\left(s\right)\right]=\Lambda_{CQG}\left(s\right).$$

The stochastic cosmological constant in Equation (46) carries a linear (i.e., odd) dependence on α, which therefore averages to zero over the symmetric Gaussian PDF. The α-averaged cosmological constant in the stochastic set $I_{r}$ therefore coincides with the expression obtained in previous papers (see [49,50]), so that no evidence of corrections to its expression due to the α-stochastic effect appears in its averaged value.

(2) The de Sitter radius: 

Let us now consider the α-average of the de Sitter radius $r_{\alpha -EH}$ given by Equation (48). In this case, the dependence on α enters through the inverse of the square-root of the stochastic cosmological constant $\Lambda \left(\alpha \right)$. Again, no dependence on Δg appears by construction, so the stochastic average only involves the Gaussian PDF on α. We have by definition:

(55) $$\begin{array}{ccc} \langle r_{\alpha -EH}\rangle & = & \int_{U_{g}}d\left(\Delta g\right)\int_{-\infty }^{+\infty }d\alpha \left[r_{\alpha -EH}\rho_{\alpha }\left(s\right)\right]=\int_{U_{g}}d\left(\Delta g\right)\int_{-\infty }^{+\infty }d\alpha \left[\sqrt{\frac{3}{\Lambda \left(\alpha \right)}}\rho_{\alpha }\left(s\right)\right]\\ & = & \sqrt{\frac{3}{\Lambda }}\int_{U_{g}}d\left(\Delta g\right)\int_{-\infty }^{+\infty }d\alpha \left[{\left(1-\alpha \right)}^{-1/2}\rho_{\alpha }\left(s\right)\right]. \end{array}$$

In order to evaluate the integral, we proceed by expanding the square-root to second order in α:

(56) $${\left(1-\alpha \right)}^{-1/2}=1+\frac{1}{2}\alpha +\frac{3}{8}\alpha^{2}+O\left(\alpha^{3}\right).$$

The linear term averages to zero, recovering the estimate for $r_{\alpha -EH}$ given by Equation (51). However, the quadratic term gives a non-vanishing contribution. In particular, neglecting the contributions of $O\left(\alpha^{3}\right)$, one obtains that:

(57) $$\begin{array}{ccc} \langle r_{\alpha -EH}\rangle & = & \sqrt{\frac{3}{\Lambda }}\int_{U_{g}}d\left(\Delta g\right)\int_{-\infty }^{+\infty }d\alpha \left[\left(1+\frac{1}{2}\alpha +\frac{3}{8}\alpha^{2}\right)\rho_{\alpha }\left(s\right)\right]\\ & = & \sqrt{\frac{3}{\Lambda }}\left(1+\frac{3}{16}\varepsilon^{2}\right)=r_{EH}\left(1+\frac{3}{16}\varepsilon^{2}\right). \end{array}$$

The particular dependence of $r_{\alpha -EH}$ on α therefore yields an average contribution to $\langle r_{\alpha -EH}\rangle$, which is of $O\left(\varepsilon^{2}\right)$. Outside the stochastic quantum belt surrounding the event horizon, this term can be regarded as negligible, but not near the de Sitter horizon. It follows that, in the same set, the averaged cosmological constant is not affected by stochastic dependence in α, while the de Sitter radius displays such a dependence. More precisely, the radius is increased by the factor $\frac{3}{16}\varepsilon^{2}$, so that the event horizon is placed further away at a greater radius, because of such a stochastic quantum gravity effect. However, remarkably, although the radius changes, the averaged curvature of the de Sitter space-time, the solution of the quantum-modified EFE, remains unchanged. This represents a purely quantum stochastic effect on the classical solution of EFE.

The consequences are that:

(a) There is a deviation of the relationship between the value of the cosmological constant and the radius of the event horizon with respect to the deterministic case in the absence of stochastic phenomena. The averaged curvature of the de Sitter universe remains the same as in the absence of α-stochastic effects, but the location of the event horizon defined by the radius of the de Sitter solution is changed.

(b) The stochastic averages of the EFE and that of the solution of EFE do not commute. As a consequence, linear stochastic contributions on the parameter α that appear in the effective cosmological constant are finally brought as non-vanishing contributions into the non-linear solution for the space-time metric tensor by the quantum-modified EFE.

(c) The solution given byEquation (57) yields a posteriori a physical interpretation of ε, which identifies the half-width of the Gaussian PDF for the distribution of the stochastic scalar α.

(d) This outcome also shows that in a cosmological scenario, the observational measure of the cosmological constant and the de Sitter radius are not equivalent, but rather they are complementary. In particular, the underlying action of stochastic effects described by the α-model outlined above can only appear in the solution of the non-linear EFE, and for the specific case of the de Sitter solution, in the value of the corresponding de Sitter radius.

(3) The Hawking temperature:

Following the discussion reported in [7], one of the critical issues of black hole dynamics is the effect of Hawking radiation on black hole evaporation, and in particular the resulting effect on the survival of primordial black holes due to Hawking evaporation in early cosmology and their possible contribution as dark matter components. In particular, one must envisage additional quantum effects (possibly operating down to the Planck scale) capable of interrupting the Hawking evaporation and make possible its final setup in the absence of singularity. Apart from this issue, that is discussed in the Introduction of the cited paper, an aspect of interest is about the possibility of defining a Hawking temperature also for a de Sitter solution. In fact, usually, the Hawking temperature is defined for classical black holes having a central singularity “hidden” by the event horizon. The de Sitter cosmological solution is singularity-free, but possesses a cosmological horizon (the boundary of the universe). As shown in [27], the Hawking temperature is associated with the emission spectrum of a black hole as seen by an observer located outside the event horizon. It is therefore formally possible to extend the definition of the Hawking temperature to the de Sitter event horizon. In the following, we shall adopt for this aim the mathematical derivation presented in [27].

More precisely, we are interested here in applying the stochastic features derived above to the estimation of the Hawking temperature for the stochastic de Sitter solution obtained retaining α effects. We start by the definition of the de Sitter temperature (see Equation (9) in [27]):

(58) $$T_{c}=-\frac{1-\Lambda r_{c}^{2}}{4\pi r_{c}},$$

where $r_{c}$ denotes the radius of the cosmological horizon in the de Sitter metric. Thus, introducing the substitutions appropriate for the stochastic de Sitter solution, namely:

$$\begin{array}{ccc} r_{c} & \to & r_{\alpha -EH},\\ \Lambda & \to & \Lambda \left(\alpha \right), \end{array}$$

we obtain a stochastic representation for the Hawking temperature, denoted here as $T_{c}\left(\alpha \right)$ and given by:

(59) $$T_{c}\left(\alpha \right)=-\frac{1-\Lambda \left(\alpha \right)r_{\alpha -EH}^{2}}{4\pi r_{\alpha -EH}}.$$

Elaborating the expression, one obtains:

(60) $$T_{c}\left(\alpha \right)=T_{c}{\left(1-\alpha \right)}^{1/2},$$

where $T_{c}$ is the customary Hawking temperature (58) of the de Sitter solution in the absence of α-stochastic effects, while the explicit contribution due to α has been singled-out.

We can therefore compute the stochastic average of such a temperature, namely $\langle T_{c}\left(\alpha \right)\rangle$. We preliminarily expand in Taylor series the square-root:

(61) $${\left(1-\alpha \right)}^{1/2}=1-\frac{1}{2}\alpha -\frac{1}{8}\alpha^{2}+O\left(\alpha^{3}\right).$$

As for the de Sitter radius, the linear term averages to zero. We obtain in fact:

(62) $$\begin{array}{ccc} \langle T_{c}\left(\alpha \right)\rangle & = & \int_{U_{g}}d\left(\Delta g\right)\int_{-\infty }^{+\infty }d\alpha \left[T_{c}\left(\alpha \right)\rho_{\alpha }\left(s\right)\right]=T_{c}\int_{U_{g}}d\left(\Delta g\right)\int_{-\infty }^{+\infty }d\alpha \left[{\left(1-\alpha \right)}^{1/2}\rho_{\alpha }\left(s\right)\right]\\ & = & T_{c}\left(1-\frac{\varepsilon^{2}}{16}\right). \end{array}$$

The result is consistent with the finding of the behavior of the de Sitter radius. In fact, the Hawking temperature is proportional to the inverse of the event horizon radius. Since the stochastically-averaged value of the radius increases, then the “surface” gravity experienced by an observer in the vicinity of the same event horizon is correspondingly reduced.

In conclusion, the stochastic quantum effects modeled by the scalar contribution in α with a given quantum PDF do not contribute to the observable average measure of the cosmological constant, but can affect nevertheless the location of the event horizon and the value of Hawking temperature. The hint that can be inferred from this calculation is that the quantum stochastic effect pointed out here due to α-dependence in the quantum gravity PDF can help decrease the black hole evaporation rate, suggesting a possible mechanism able to permit the survival of primordial black holes. The physical scenario depicted here involves uniquely the stochastic quantum behavior of the gravitational field without requiring the action of any additional field, and for this reason, it is a purely quantum effect of the gravitational field.

## 6. Conclusions

In this paper, we showed that in the framework of a stochastic and hydrodynamic approach to the manifestly-covariant quantum gravity theory, the quantum-produced cosmological constant arising due to collective vacuum graviton interactions (Bohm interaction) can in principle carry stochastic corrections, just as the background field tensor. The basic conceptual implication is that regions with different metric signatures, which are separated by an event horizon, are no longer treated as strictly impenetrable both for quantum particles and classical matter. This concerns in principle the occurrence of a novel tunneling mechanism arising across event horizons, which combines quantum gravity with the stochastic properties of the quantum gravitational field and its modification of classical metric tensor solutions. The case study of cosmological de Sitter solution has been treated. According to the present outcome in fact, it is the classical de Sitter event horizon that acquires a quantum and stochastic character, so that it no longer remains defined as a deterministic barrier, fixed at a prescribed classical radius. Rather, the event horizon acquires a quantum width compatible with the occurrence of tunneling effects and still satisfying the unitarity principle of quantum mechanics. These features arise from the solution of the quantum continuity equation associated with the quantum gravity wave equation and the inspection of indeterminacy properties for the inclusion of stochastic parameters in the solution of the quantum probability density, which can preserve its Gaussian profile. Observational consequences on the calculation of the radius of the de Sitter event horizon and related Hawking temperature were discussed. The conceptual implications of the solution considered in the paper are challenging, involving the possible generation and emission of massive gravitons, particle or fields across the de Sitter event horizon, as well as indicating the possible occurrence of similar effects for other (classical) black hole solutions. The conclusions reached here are therefore meaningful in the context of quantum gravity, theoretical astrophysics, and cosmology for their potential physical relevance concerning the characterization of quantum phenomena and tunneling effects that can occur in the surroundings of event horizons.
